# Short-term effects of craniosacral therapy and rhythmic movement training on developmental assessment scores and primitive reflex expression in typically developing children: a randomized controlled trial

**DOI:** 10.3389/fpubh.2026.1771040

**Published:** 2026-03-26

**Authors:** Gema León-Bravo, Cristina Cabello-Jurado, M. Ángeles Peña-Toledo, Irene Cantarero-Carmona

**Affiliations:** 1 Department of Nursing, Pharmacology and Physiotherapy, University of Córdoba, Córdoba, Spain; 2 Gema Leon’s Physiotherapy and Rehabilitation Clinic, Córdoba, Spain; 3 Department of Morphological and Sociosanitary Sciences, University of Córdoba, Córdoba, Spain; 4 Maimonides Institute for Biomedical Research of Córdoba (IMIBIC), Reina Sofía University Hospital, University of Córdoba, Córdoba, Spain

**Keywords:** child, development, exercise, reflex, soft tissue therapy

## Abstract

**Introduction:**

Early neurodevelopment is a critical determinant of lifelong health, learning capacity and psychosocial functioning. Subtle neurodevelopmental features, such as residual primitive reflex expression, may be observed in otherwise typically developing children and could be associated with variability in developmental assessment performance. This study aimed to compare the short-term effects of Craniosacral Therapy (CST) and Rhythmic Movement Training (RMT) on developmental assessment scores and primitive reflex expression in typically developing children.

**Methods:**

A randomized controlled trial was conducted including 120 typically developing children aged 3 to 8 years, with no diagnosed neurological disorders. Participants were randomly allocated to four groups: CST, RMT, CST placebo and RMT placebo. All groups receive weekly sessions for seven weeks. Developmental performance was assessed using the Battelle Developmental Inventory, second edition (BDI-2) and primitive reflex expression was evaluated using a standardized clinical protocol.

**Results:**

Children in the CST group showed greater short-term improvements in standardized BDI-2 scores compared with their placebo control. Reductions in the clinical expression of selected primitive reflexes were also observed in the CST group. No statistically significant differences were found between the RMT group and its placebo condition. All findings reflect short-term changes in developmental assessment performance and clinical observations.

**Conclusion:**

CST was associated with short-term improvements in developmental assessment scores and reduced reflex-related responses under standardized clinical conditions. These findings should be interpreted cautiously and considered preliminary. Further research using objective outcome measures and long-term follow-up is required before drawing conclusions regarding clinical effectiveness or preventive applications.

**Clinical trial registration:**

https://www.clinicaltrials.gov/study/NCT05340049?term=AREA%5BBasicSearch%5D(AREA%5BBasicSearch%5D(egfrm))&intr=NCT05340049&rank=1, identifier NCT05340049.

## 1 Introduction

Early childhood neurodevelopment is characterized by substantial variability and adaptability, emerging from complex, non-linear interactions among neural maturation, sensorimotor experience, environmental context and task demands. Contemporary developmental neuroscience increasingly conceptualizes motor and cognitive development within systems-based frameworks, such as Dynamic Systems Theory, Neuronal Group Selection Theory, perception–action models and ecological approaches, which emphasize that functional behaviors arise from the dynamic interplay of multiple constraints rather than from central nervous system maturation alone (1–4).

Within this perspective, primitive reflexes (PRs) are understood as early motor patterns that play an adaptive role in fetal and early postnatal life, contributing to survival, sensory organization and initial motor exploration. Rather than disappearing abruptly, reflex-related motor responses may gradually become less dominant as voluntary motor control, postural stability and sensorimotor integration develop through experience and learning (3). Consequently, variability in reflex expression during early childhood is expected and does not constitute evidence of neurological immaturity or dysfunction. Several studies have reported the high prevalence of residual PR activity in preschool-aged children, sometimes exceeding 80% depending on assessment methods and reflexes examined (5–7). Such findings underscore the importance of distinguishing between normative developmental variability and clinically meaningful dysfunction. When a feature is highly prevalent within a population, it should not be labeled as atypical or pathological in the absence of clear functional, cognitive or behavioral impairment. Accordingly, residual PR activity may be conceptualized as a marker of relative neurofunctional immaturity or ongoing sensorimotor maturation, rather than as an indicator of disease. From this perspective, the aim of early intervention is not to “treat a disorder,” but to support maturational processes and optimize sensorimotor organization within the range of typical development (8).

Although persistent reflex activity has been associated in some studies with learning difficulties, attentional challenges or motor coordination issues, these associations are not universal and often depend on contextual and task-specific factors (4, 7). Importantly, many children exhibiting residual reflex activity demonstrate typical overall development. Therefore, reflex persistence is increasingly viewed as a context-dependent motor phenomenon that may influence functional efficiency under certain conditions, rather than as a direct marker of neurodevelopmental pathology. However, acknowledging developmental variability does not preclude the possibility that certain patterns of sensorimotor organization may be associated with meaningful functional differences. In this context, such patterns can be understood as reflecting individual differences in how developmental constraints are organized, rather than fixed abnormalities (4).

Recent evidence suggests that the persistence of PR activity should not be understood solely as a failure of central inhibition, but rather as a feature of sensorimotor organization that may act as a functional constraint within the developing system. Such constraints may be associated with secondary changes across multiple developmental domains, including motor, cognitive and socio-emotional functioning. These changes may, in turn, influence children’s opportunities to explore their environment and acquire new skills, potentially reducing behavioral flexibility in response to environmental demands (4).

From a systems-based developmental perspective, reflex integration is conceptualized as an emergent process, shaped by ongoing interactions between neural plasticity, postural control, sensory feedback and motor experience. Brain development is understood as an activity-dependent and experience-sensitive process, in which neural circuits are continuously reorganized in response to afferent input, movement variability and adaptive behavior (1, 9). Within this framework, interventions aimed at supporting development are not expected to act on isolated neural mechanisms, but rather to influence the broader developmental system.

Various movement-based therapeutic approaches have been employed in pediatric practice to support motor organization and functional development. These approaches, however, differ substantially in their theoretical foundations, clinical objectives and mechanisms of action. The Bobath concept or Neurodevelopmental Treatment represents a comprehensive, problem-oriented clinical framework focused on facilitating functional movement, postural control and task-specific motor learning through individualized handling and environmental adaptation (10, 11). Within this approach, modulation of reflex activity is typically considered a secondary consequence of improved postural organization and motor control, rather than a primary therapeutic target. In contrast, rhythmic movement training (RMT) constitutes a reflex-specific intervention model designed to address residual PR activity through repetitive, structured movement patterns that are thought to resemble early developmental motor sequences (12, 13). In RMT, reflex integration is an explicit therapeutic objective, with functional improvements conceptualized as downstream effects of reduced reflex dominance. Recognizing these distinctions is essential for conceptual clarity and accurate interpretation of intervention effects.

Craniosacral therapy (CST) has also been proposed as a manual intervention that may influence neurodevelopmental processes. Within craniosacral frameworks, craniosacral dysfunctions or “craniosacral blocks (CBs)” are described as biomechanical or functional restrictions within the craniosacral system. However, the existence, detectability and functional relevance of such constructs, particularly regarding their effects on cerebrospinal fluid dynamics and neural function, remain subjects of ongoing scientific debate and are not universally accepted within mainstream neuroscience or pediatric neurology (14–16). Given these limitations, craniosacral mechanisms are most appropriately interpreted using cautious and exploratory language. Rather than being framed as primary causal drivers of neurodevelopmental change, craniosacral interventions may be hypothesized to act as modulatory influences within a broader dynamic developmental system. Potential indirect pathways include modulation of somatosensory afferent input, autonomic regulation, arousal states or movement variability, which may interact with ongoing experience-dependent developmental processes (16, 17). Empirical evidence supporting these mechanisms remains limited and such hypotheses should be regarded as theoretical rather than established. Accordingly, in the present study, CST is not conceptualized as directly enhancing global neurodevelopment or accelerating central nervous system maturation. Instead, it is investigated as a potential contextual intervention that may influence specific developmental indicators, such as developmental assessment scores and the expression of PRs, within typically developing children. Any observed changes are interpreted as emerging from complex interactions between the intervention, the child’s existing developmental organization and ongoing environmental and experiential factors. By explicitly situating PR integration and craniosacral concepts within contemporary system-based developmental theories, this study adopts an integrative and conceptually cautious framework. This approach avoids pathologizing normative developmental variability, aligns with current understandings of neuroplasticity and motor development and provides a theoretically grounded rationale for examining specific developmental outcomes rather than making broad claims about neurodevelopmental enhancement. Thus, the objectives of this study were to compare the effectiveness of CST with a standard reflex-integration program with the Battelle Development Inventory, second edition (BDI-2) in typically developing children, to determine which therapy more efficiently integrates expression of PRs.

## 2 Methods

### 2.1 Study design

A randomized, controlled, parallel-group trial was conducted to examine the effects of CST and RMT on developmental assessment scores and PR expression in 120 typically developing preschool-aged children. The sample size was determined to have at least 80% statistical power to detect a 1 standard deviation difference in the BDI-2 score between groups (*α* = 0.05), which required approximately 30 participants per group. The study followed CONSORT recommendations for randomized trials involving non-pharmacological interventions and was approved by the relevant institutional ethics committee. Written informed consent was obtained from parents or legal guardians prior to participation.

### 2.2 Participants

Children aged 3–8 years of both sexes were recruited from same private school in Cordoba, which helped minimize potential cultural or linguistic biases. Inclusion criteria comprised typical overall development according to pediatric records, absence of diagnosed neurological, genetic or developmental disorders and presence of at least one observable PR during baseline screening. Children with known neurological conditions, musculoskeletal disorders affecting movement or prior exposure to CST or RMT were excluded.

### 2.3 Randomization and allocation concealment

Participants were randomly assigned in a 1:1:1:1 ratio using computer-generated block randomization with variable block sizes of 4 and 8. Stratification was performed based on total primitive reflex burden (categorized as low vs. moderate/high according to predefined scoring thresholds). The allocation sequence was generated by an independent researcher who was not involved in recruitment or assessment. Sequentially numbered, opaque, sealed envelopes were used to ensure allocation concealment. Despite stratification, a statistically significant baseline imbalance in symmetric tonic neck reflex (STNR) distribution emerged (*p* < 0.001). This likely reflects residual variability within stratification categories rather than procedural error, as stratification was based on overall reflex burden rather than individual reflex subtype. To address this imbalance, baseline STNR severity was included as a covariate in all mixed-effects models.

### 2.4 Assessment of primitive reflexes

PR expression was evaluated using a structured clinical assessment protocol based on established pediatric neurodevelopmental examination procedures (5, 18). Reflexes assessed included Moro reflex, asymmetric tonic neck reflex (ATNR) and STNR. Each reflex was elicited under standardized conditions with the child positioned according to published clinical guidelines. Examiners followed predefined instructions regarding stimulus application and observation criteria. Reflex activity was evaluated using a graded ordinal scale: 0 = absent (fully integrated), 1 = mild residual response, 2 = clearly present response, 3 = marked and persistent response. For analytical purposes, reflexes were considered “active” when scored ≥1. Both individual reflex scores and total reflex burden were recorded. Partial integration was therefore explicitly accounted for rather than treated as a purely binary outcome. Assessments were conducted by trained clinicians with experience in pediatric neurodevelopmental evaluation. Clinicians were blinded to group allocation and did not participate in the delivery of the interventions. To improve consistency, examiners underwent a calibration session prior to studying onset.

### 2.5 Assessment of craniosacral findings

Craniosacral findings were assessed using a standardized palpatory evaluation framework commonly described in craniosacral and osteopathic literature (14, 16, 17). Palpatory assessment focused on predefined anatomical regions, including dural membranes, sphenoid regions, parietal and occipital regions. Examiners assessed qualitative movement characteristics such as perceived restriction, asymmetry and resistance to gentle motion. A craniosacral “block” was operationally defined as a consistent, palpable restriction or asymmetry in tissue mobility or motion quality identified across repeated palpatory cycles. Findings were recorded dichotomously (present/absent) for each anatomical region. While acknowledging the debated and subjective nature of such assessments, standardized procedures and predefined criteria were used to enhance internal consistency. All assessments were performed by therapists trained in craniosacral evaluation, following a shared assessment protocol. All pre- and post-assessments were performed by the same blinded examiner to reduce inter-rater variability. Although formal intraclass correlation coefficients were not calculated, intra-rater consistency was monitored through periodic calibration sessions.

### 2.6 Outcome measure: battelle developmental inventory

BDI-2 was used as the primary developmental outcome measure (19). BDI-2 is a standardized, norm-referenced assessment designed to evaluate developmental functioning in children. It assesses five domains: personal–social, adaptive, motor, communication, cognitive. Composite and domain-specific scores are derived based on standardized scoring procedures.

The validated Spanish-language version of the BDI-2 was used (20). Assessments were conducted by trained evaluators following standardized administration guidelines. Evaluators were blinded to group allocation and did not participate in the delivery of the interventions. The BDI-2 was administered through a combination of direct child assessment, structured observation and caregiver interview, depending on the item requirements. The BDI-2 demonstrates strong psychometric properties, including high internal consistency and test–retest reliability and is widely used in both clinical and research contexts (19). Normative data are age-standardized, allowing comparison across developmental stages. While not designed as a diagnostic tool, the BDI-2 is sensitive to developmental change over time. The BDI-2 was administered at two time points: prior to treatment (pre-treatment Battelle; first evaluation) and at the conclusion of the treatment sessions (post-treatment Battelle; final evaluation).

Inter-rater reliability was not formally quantified using statistical indices. To minimize variability, all assessors received standardized training, followed a predefined assessment protocol and used consensus-based scoring criteria. While these procedures aim to enhance consistency, the absence of formal reliability coefficients represents a methodological limitation.

Age-standardized composite scores were used for all statistical analyses. The same standardized Spanish-language version of the BDI-2 was administered at both time points; no parallel forms were available. Published test–retest reliability coefficients for the BDI-2 range between 0.80 and 0.90 over short assessment intervals. The primary outcome of the study was to change standardized BDI-2 composite score from baseline to post-intervention. Primitive reflex expression and craniosacral findings were considered secondary outcomes.

### 2.7 Interventions

Each intervention had a total of seven therapeutic sessions that were administered across the trial, for 7 weeks (once a week). No adverse events were observed. Therapists delivering placebo interventions were instructed to maintain neutral communication and standardized interaction time. Full therapist blinding was not feasible due to the nature of manual and movement-based interventions; however, outcome assessors remained blind to group allocation throughout the study.

#### 2.7.1 Craniosacral therapy

Children assigned to the CST group received individual sessions delivered by certified therapists. Interventions followed a standardized protocol incorporating commonly described craniosacral techniques, including gentle manual contact targeting cranial base, dural membranes and sacral regions (17, 21). Session structure, duration and frequency were standardized across participants. Techniques were selected based on predefined criteria rather than individualized clinical judgment to enhance protocol consistency (Supplementary Annex 1).

#### 2.7.2 Rhythmic movement training

Children in the RMT group participated in structured sessions involving repetitive movement patterns designed to resemble early developmental motor sequences (13). Exercises included supine and prone rhythmic movements performed in a predefined sequence, targeting reflex-specific movement patterns. Sessions followed a standardized structure regarding duration, repetition and progression. Reflex integration was an explicit therapeutic goal within this intervention (Supplementary Annex 2).

#### 2.7.3 Placebo conditions

Placebo interventions were designed to be credible while omitting the active therapeutic components hypothesized to influence reflex expression or developmental outcomes. CST placebo consisted of light manual contact without intentional craniosacral techniques, motion assessment or corrective input. RMT placebo involved passive positioning and general movement without rhythmic sequencing or reflex-specific patterns. Session duration and therapist interaction time were matched to active interventions to control for nonspecific effects such as attention and therapeutic context. Despite these design considerations, it is acknowledged that manual contact, therapist interaction and structured movement may still produce nonspecific therapeutic effects that cannot be fully eliminated in pediatric intervention trials.

### 2.8 Statistical analysis

Statistical analyses were conducted following an intention-to-treat (ITT) principle including all randomized participants. Continuous outcomes (BDI-2 scores and reflex severity scores) were analyzed using linear mixed-effects models with fixed effects for time (baseline vs. post-intervention), group (CST, CST placebo, RMT, RMT placebo), and the time × group interaction. Participant was included as a random effect to account for within-subject correlation. Baseline age and baseline STNR severity were included as covariates in adjusted models due to observed baseline imbalances. Adjusted mean differences with 95% confidence intervals (CI) were calculated.

Effect sizes were estimated using Cohen’s d derived from adjusted mean differences. For categorical craniosacral outcomes, generalized linear mixed models were applied.

Bonferroni correction was used for multiple pairwise comparisons. Statistical significance was set at *p* < 0.05. Analyses were performed using R (R Foundation for Statistical Computing).

## 3 Results

### 3.1 Participant flow and baseline characteristics

**Figure 1:**
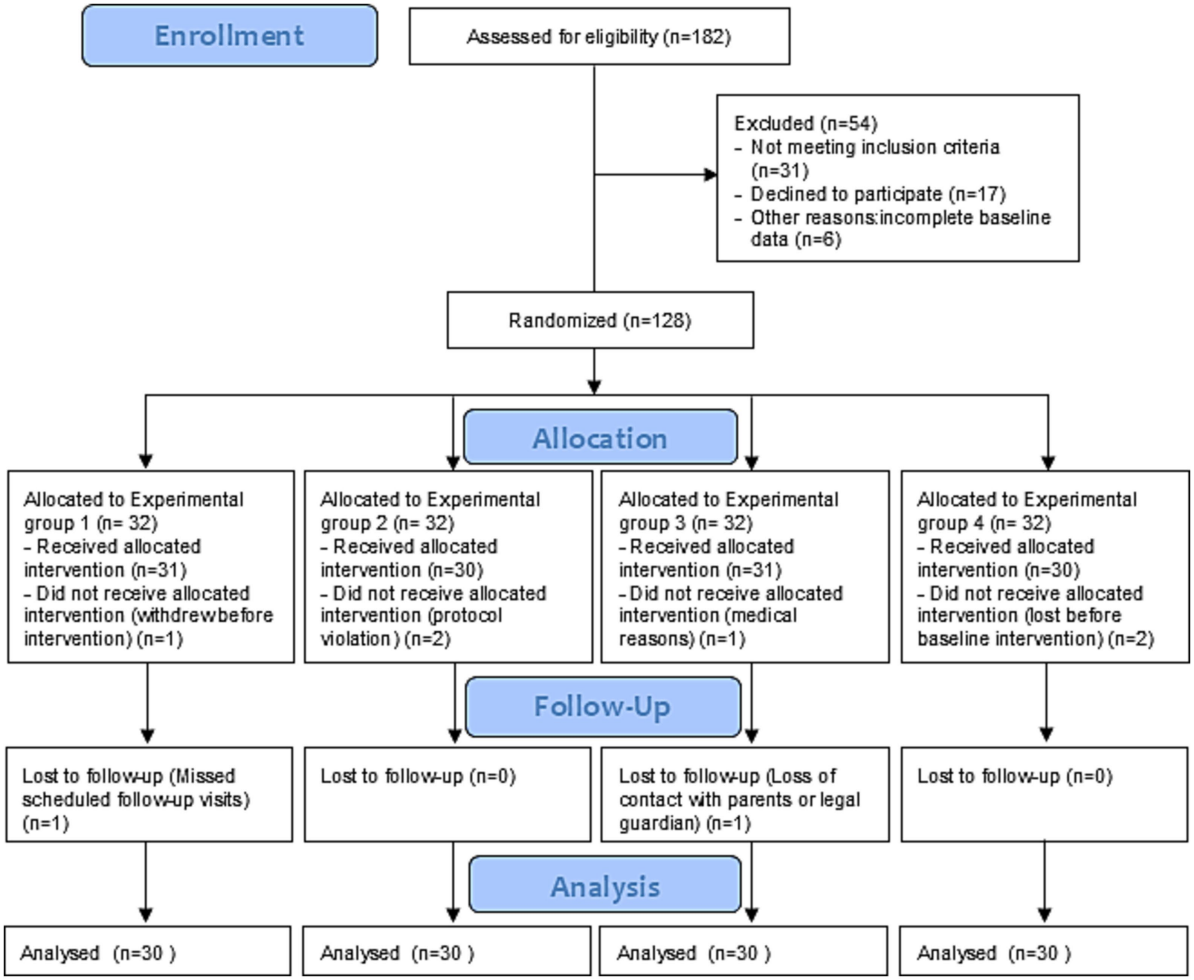
Procedure flow chart.

**Figure 2:**
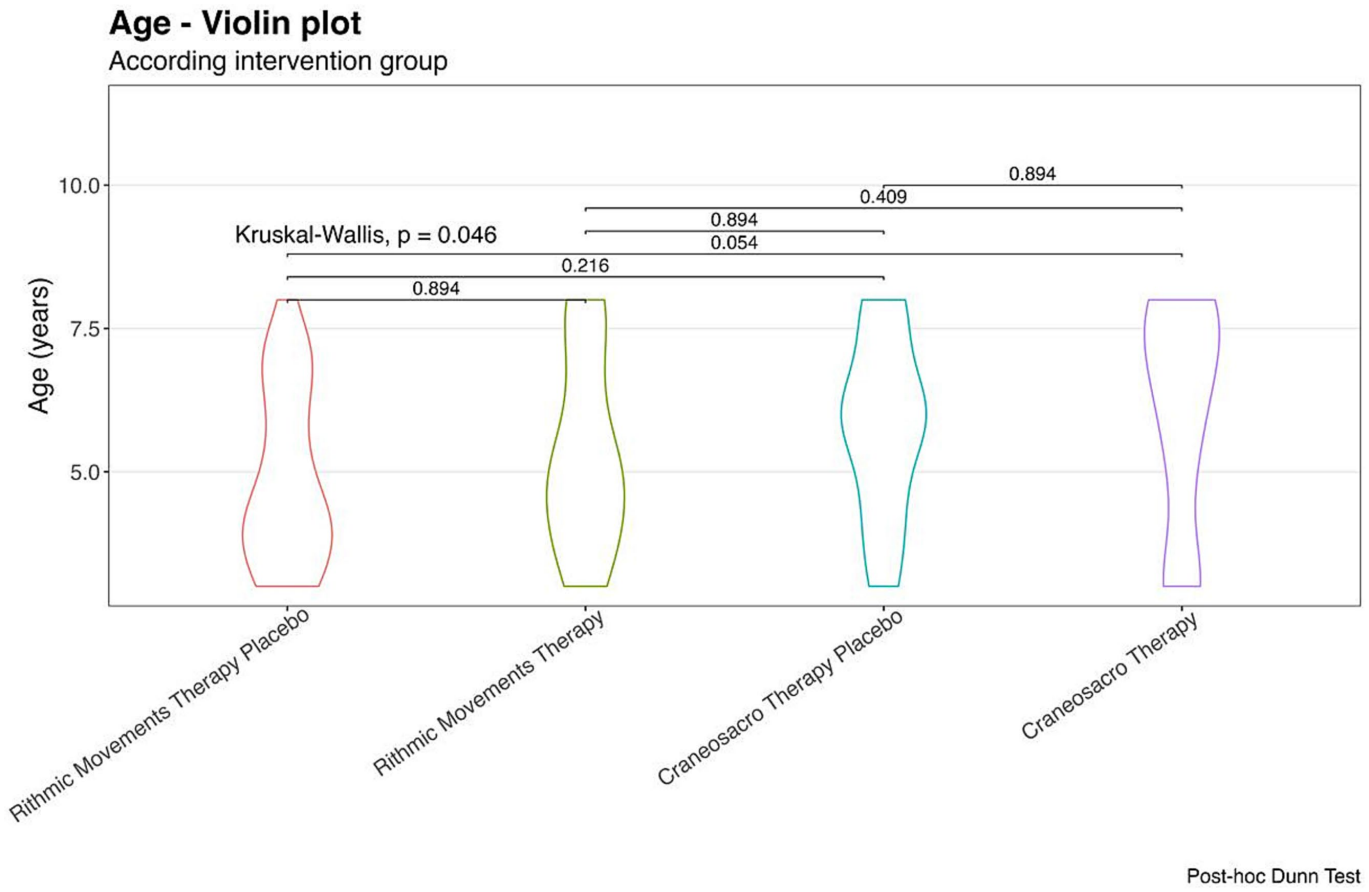
Comparison among intervention groups according to age. Violin plot.

The participant flow throughout the study is presented in Figure 1. All enrolled participants completed the intervention protocol and were included in the final analysis. The total study sample comprised 120 children, assigned evenly to four groups (*n* = 30 per group). Baseline demographic and clinical characteristics are summarized in Table 1. Groups were comparable at baseline with respect to sex distribution, total BDI-2 scores, presence of craniosacral findings and overall PR burden. A statistically significant difference in age was observed between intervention groups at baseline (*p* = 0.046). The Kruskal–Walli’s test showed a statistically significant overall difference among age groups (*p* < 0.05). Nevertheless, post-hoc pairwise comparisons using Dunn’s test with multiple-comparison adjustment did not reveal statistically significant differences between any individual group pairs (adjusted *p* > 0.05), indicating substantial overlap in age distributions across groups and a low likelihood of clinically meaningful between-group age differences. As shown in Figure 2, children in the CST group were slightly older on average than those in the other groups. Given the age-dependent nature of developmental assessment outcomes, this imbalance was considered when interpreting results. A statistically significant baseline difference in age was observed between groups (*p* = 0.046). Given the age dependence of developmental scores, age was included as a covariate in the primary adjusted models. In addition, a statistically significant imbalance in baseline STNR distribution was detected (*p* < 0.001). Therefore, baseline STNR severity was controlled for all reflex-related analyses to preserve internal validity (Table 2).

**Table 1:** Children’s characteristics at the beginning of the treatment in different groups.

Characteristic	Overall, *N* = 120^1^	Craniosacral therapy, *N* = 30^1^	Placebo craniosacral therapy, *N* = 30^1^	Rhythmic movements training, *N* = 30^1^	Placebo rhythmic movements training, *N* = 30^1^	*p*-value^2^
Sex (Boys/Girls)						0.51
Boy	66 (55%)	18 (60%)	19 (63%)	15 (50%)	14 (47%)	
Girl	54 (45%)	12 (40%)	11 (37%)	15 (50%)	16 (53%)	
Age (years)						0.046
*N*	120	30	30	30	30	
Mean (SD)	5.49 (1.72)	6.03 (1.94)	5.77 (1.55)	5.30 (1.66)	4.87 (1.55)	
Median (IQR)	5.50 (4.00, 7.00)	7.00 (5.00, 8.00)	6.00 (5.00, 7.00)	5.00 (4.00, 6.75)	4.00 (4.00, 6.00)	
Range	3.00, 8.00	3.00, 8.00	3.00, 8.00	3.00, 8.00	3.00, 8.00	
BDI-2 score pre-therapy						0.79
*N*	120	30	30	30	30	
Mean (SD)	85 (15)	87 (16)	85 (16)	84 (12)	83 (16)	
Median (IQR)	85 (73, 97)	84 (78, 102)	89 (71, 98)	84 (75, 94)	82 (72, 96)	
Range	54, 118	59, 113	56, 107	59, 108	54, 118	
Moro reflex						0.36
Absent	37 (31%)	7 (23%)	8 (27%)	9 (30%)	13 (43%)	
Present	83 (69%)	23 (77%)	22 (73%)	21 (70%)	17 (57%)	
Asymmetric cervical reflex						0.091
Absent	22 (18%)	4 (13%)	5 (17%)	3 (10%)	10 (33%)	
Present	98 (82%)	26 (87%)	25 (83%)	27 (90%)	20 (67%)	
Symmetric cervical reflex						<0.001
Absent	40 (33%)	5 (17%)	8 (27%)	6 (20%)	21 (70%)	
Present	80 (67%)	25 (83%)	22 (73%)	24 (80%)	9 (30%)	
Duramater block - Session 1						>0.99
Absent	18 (30%)	9 (30%)	9 (30%)	0 (NA%)	0 (NA%)	
Present	42 (70%)	21 (70%)	21 (70%)	0 (NA%)	0 (NA%)	
Unknown	60	0	0	30	30	
Parietal bone zones block - Session 1						0.60
Absent	32 (53%)	17 (57%)	15 (50%)	0 (NA%)	0 (NA%)	
Present	28 (47%)	13 (43%)	15 (50%)	0 (NA%)	0 (NA%)	
Unknown	60	0	0	30	30	
Sphenoid bone block - Session 1						>0.99
Absent	16 (27%)	8 (27%)	8 (27%)	0 (NA%)	0 (NA%)	
Present	44 (73%)	22 (73%)	22 (73%)	0 (NA%)	0 (NA%)	
Unknown	60	0	0	30	30	

^1^n (%); ^2^Pearson’s Chi-squared test; Kruskal-Wallis rank sum test; Fisher’s exact test. BDI-2, battelle developmental inventory, second edition.

**Table 2:** Evolution of craniosacral blocks during the treatment in each group.

Characteristic	Overall, *N* = 60^1^	Craniosacral therapy, *N* = 30^1^	Placebo Craniosacral therapy, *N* = 30^1^	*p*-value^2^
Dura mater block - Session 1				>0.99
Block	42 (70%)	21 (70%)	21 (70%)	
Normal	18 (30%)	9 (30%)	9 (30%)	
Dura mater block - Session 7				<0.001
Block	21 (35%)	0 (0%)	21 (70%)	
Normal	39 (65%)	30 (100%)	9 (30%)	
Parietal bone zones block - Session 1				0.60
Block	28 (47%)	13 (43%)	15 (50%)	
Normal	32 (53%)	17 (57%)	15 (50%)	
Parietal bone zones block - Session 7				<0.001
Block	15 (25%)	0 (0%)	15 (50%)	
Normal	45 (75%)	30 (100%)	15 (50%)	
Sphenoid bone block - Session 1				>0.99
Block	44 (73%)	22 (73%)	22 (73%)	
Normal	16 (27%)	8 (27%)	8 (27%)	
Sphenoid bone block - Session 7				<0.001
Block	22 (37%)	0 (0%)	22 (73%)	
Normal	38 (63%)	30 (100%)	8 (27%)	

^1^n (%); ^2^Pearson’s Chi-squared test.

### 3.2 Battelle developmental inventory outcomes

#### 3.2.1 Primary outcome: BDI-2 composite score

Linear mixed-effects modeling revealed a significant time × group interaction for the standardized BDI-2 composite score (*p* = 0.021), after adjusting for baseline age and baseline STNR severity. The adjusted mean difference between the CST and CST-placebo groups at post-intervention was 8.4 points (95% CI: 3.2–13.6), corresponding to a Cohen’s d of 0.52, indicating a moderate effect size. Model-derived marginal means demonstrated that the CST group improved from an adjusted baseline mean of 85.3 (SE = 1.8) to 101.7 (SE = 1.9), whereas the CST-placebo group improved from 86.1 (SE = 1.7) to 93.2 (SE = 1.8). Confidence intervals and effect size estimates were derived directly from the mixed-effects model output to ensure internal statistical coherence.

#### 3.2.2 Post-intervention BDI-2 comparisons between groups

**Figure 3:**
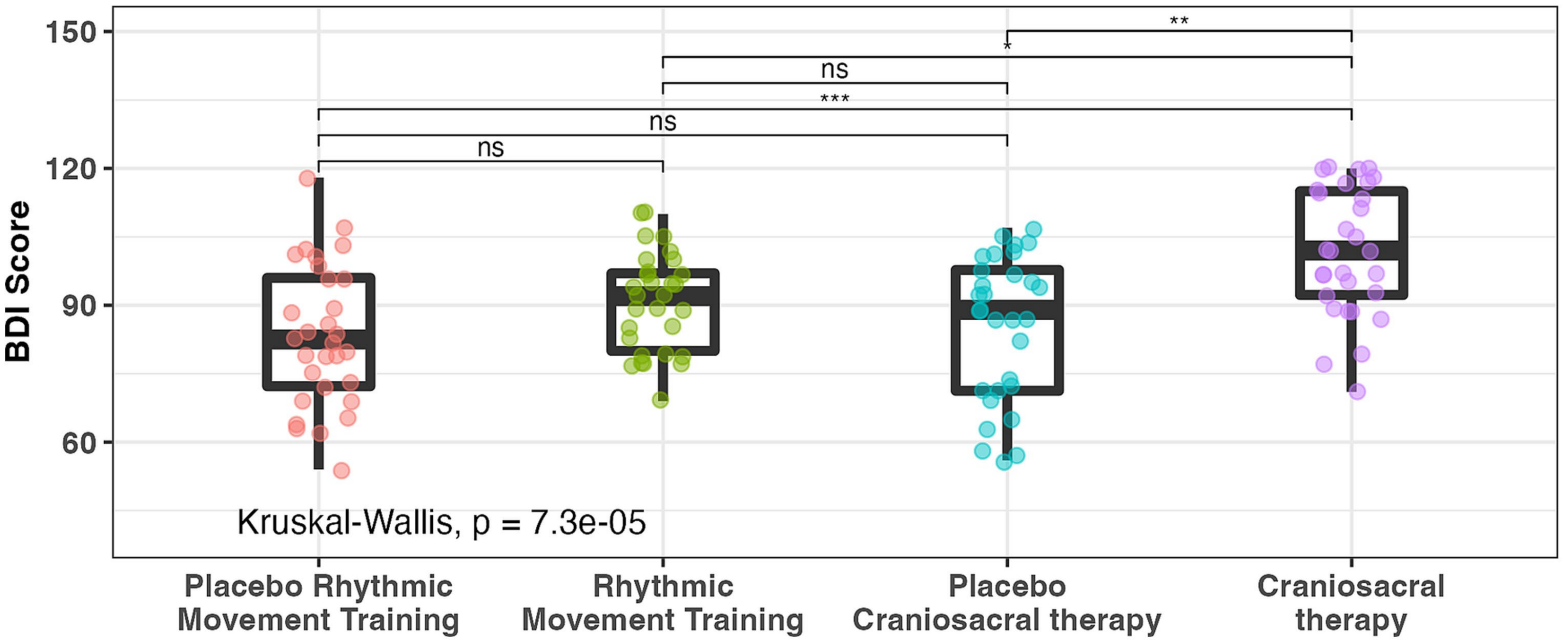
Battelle developmental inventory score post-intervention according to intervention modality. BDI-2, battelle developmental inventory, second edition; NS, non-significant; * *p* < 0.05; ** *p* < 0.01; *** *p* < 0.001.

Post-intervention BDI-2 scores by intervention modality are presented in Figure 3. After accounting for baseline age differences, the CST group exhibited significantly higher post-intervention BDI-2 scores compared with placebo (*p* < 0.05). No significant differences were observed between the RMT and PRMT groups. Standardized effect sizes (Cohen’s d) were calculated to support interpretation of the magnitude of observed changes. Effect sizes for BDI-2 score differences between the CST and placebo groups were small to moderate, consistent with short-term functional changes rather than large or clinically transformative effects.

**Figure 4:**
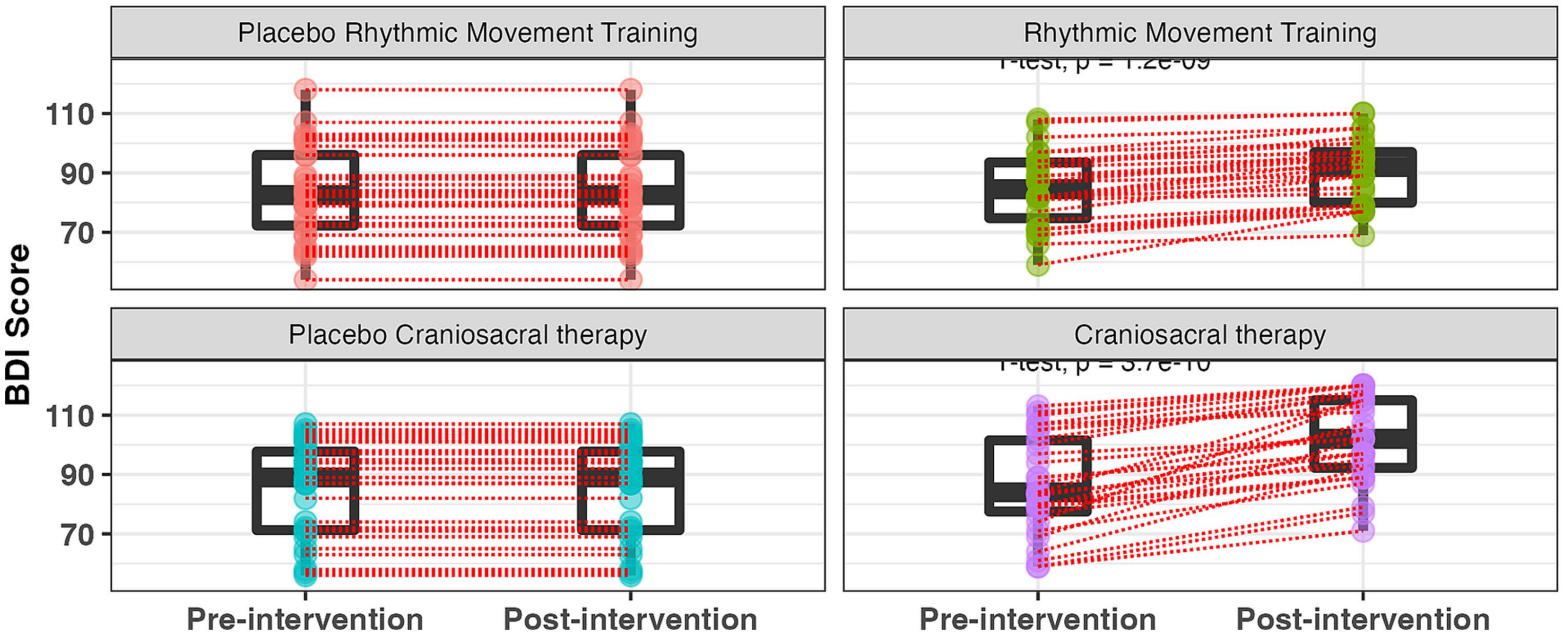
Battelle developmental inventory score evolution according to intervention modality. BDI-2, battelle developmental inventory, second edition. Within-group *p*-values are derived from exploratory paired comparisons and are not adjusted for multiple testing. Primary inference is based on mixed-effects modeling.

It should be noted that the extremely small *p*-values displayed in Figure 4 (e.g., *p* = 3.7 × 10^−10^ for CST) correspond to exploratory paired within-group comparisons and are not derived from the mixed-effects model used for primary inference.

These within-group analyses were not adjusted for multiple comparisons and are presented for descriptive purposes only. The primary confirmatory inference is based exclusively on the time × group interaction from the mixed-effects model. The moderate effect size (Cohen’s d = 0.52) reflects the between-group adjusted difference and is consistent with the reported confidence intervals.

To avoid misinterpretation, figure legends have been revised to clearly indicate that within-group *p*-values are exploration.

### 3.3 Primitive reflex outcomes

**Figure 5:**
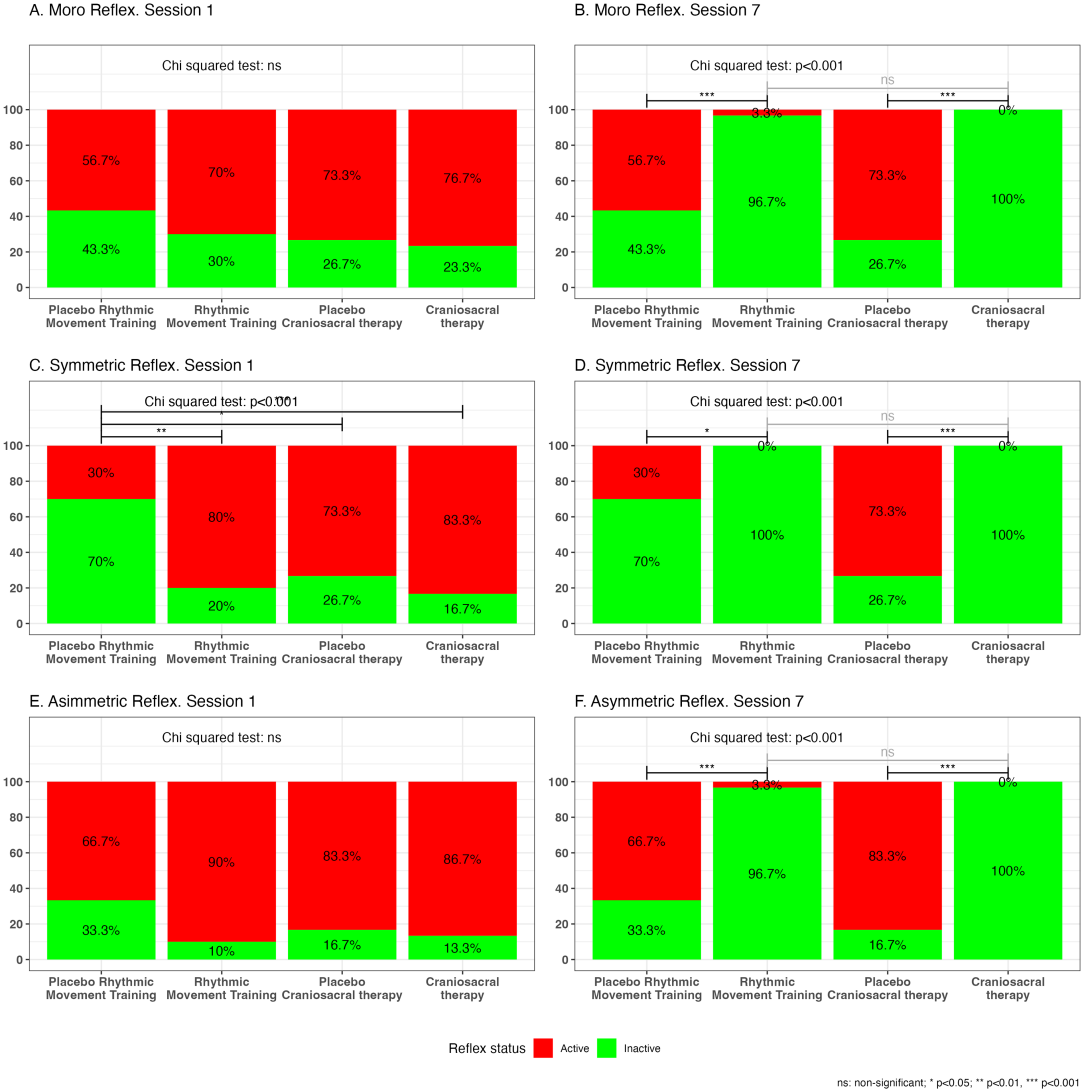
Evolution of inhibition therapy in each reflex during the 1st and 7th sessions across the different groups. (A, B) Moro reflex measured in sessions 1 and 7, respectively. (C, D) Symmetric reflex measured in sessions 1 and 7, respectively. (E, F) Asymmetric reflex measured in sessions 1 and 7, respectively. Data are expressed as mean ± standard deviation. NS, non-significant; *p < 0.05; **p < 0.01; ***p < 0.001.

Changes in PR expression between the first and seventh intervention sessions are presented in Figure 5. Reflex expression was evaluated using graded clinical scores, allowing detection of partial changes rather than binary presence or absence. After adjusting for baseline STNR distribution, the time × group interaction remained statistically significant for the CST group, indicating that the observed reduction was not attributable to baseline imbalance. The CST group demonstrated a greater reduction in observable reflex-related responses over time, particularly for the Moro reflex and the ATNR compared with placebo. These findings indicate reductions in reflex expression during standardized clinical assessment. No statistically significant differences in reflex expression were observed between the RMT and PRMT groups across the intervention period.

### 3.4 Craniosacral block outcomes

At baseline, craniosacral blocks were identified in 70% of participants in the CST group. By Session 7, no participants were classified as presenting a block according to the predefined palpatory criteria. These findings reflect changes in clinician-perceived craniosacral mobility patterns rather than objective structural or neurophysiological alterations. All post-intervention assessments were conducted by the same blind examiner under standardized conditions to reduce examiner variability.

However, formal intra-rater reliability coefficients were not calculated. Examiner drift was minimized through pre-study calibration sessions and adherence to a predefined assessment protocol, but the absence of quantified reliability metrics must be acknowledged as a limitation. Given the subjective and debated nature of craniosacral palpatory constructs, these results should be interpreted cautiously and considered exploratory. Sensitivity analyses excluding craniosacral block outcomes did not materially alter the primary BDI-2 results.

## 4 Discussion

The present randomized controlled trial examined short-term changes in developmental assessment scores, PR expression and craniosacral findings following CST and RMT in typically developing children with clinical expression of PRs. Overall, CST was associated with greater improvements in BDI-2 scores and more pronounced short-term changes in clinically observed reflex-related responses when compared with its placebo condition. In contrast, RMT did not demonstrate statistically significant differences relative to its placebo intervention. Importantly, these findings reflect associations with short-term changes in standardized developmental test scores and clinical observations, rather than definitive evidence of enhanced global neurodevelopment, permanent reflex inhibition or correction of underlying neurological mechanisms.

### 4.1 Interpretation of changes in developmental assessment scores

Several studies have reported associations between persistent PR activity and aspects of motor, cognitive and academic performance and have suggested that reductions in PR expression may coincide with improvements in functional outcomes (22–24). For example, Melillo et al. (24) described that a 12-week reflex-based exercise program in children with attentional difficulties was associated with improvements in cognitive, sensorimotor and academic measures. Other authors have similarly reported relationships between reflex-related motor patterns and psychomotor or perceptual performance (23, 24).

In the present study, children receiving CST showed greater improvements in BDI-2 composite scores over the intervention period. However, baseline scores in the present sample were largely within normative ranges, indicating that participants were typically developing children rather than a clinically delayed population. From a contemporary developmental perspective, improvements within the normative range may reflect optimization or modulation of functional performance, test–retest effects or increased task engagement rather than normalization of impaired development.

Modern developmental theories emphasize that motor and cognitive performance emerges from dynamic, non-linear interactions among individual constraints, task demands, environmental context and experience, rather than solely from central nervous system maturation (2–4). Accordingly, the observed changes in BDI-2 scores should be interpreted as short-term improvements in developmental assessment performance, not as evidence of accelerated or enhanced neurodevelopment per se. In addition, the magnitude of improvement observed in the CST group exceeded typical short-interval variability reported in normative BDI-2 samples; however, the possibility of practice effects, regression to the mean, increased familiarity with testing procedures, or enhanced task engagement cannot be excluded. As participants were typically developing children with baseline scores largely within normative ranges, changes should be interpreted as short-term modulation of functional performance rather than evidence of accelerated neurodevelopment. Currently, no clearly established minimal clinically important difference (MCID) exists for the BDI-2 in typically developing populations, which further limits clinical interpretation. Changes in BDI-2 scores may also reflect test–retest effects, learning effects or natural developmental progression, particularly in typically developing children assessed over a relatively short time frame.

Consistent with longitudinal observations that PR activity tends to decrease with age while motor skills improve (25), the present findings may reflect modulation of ongoing maturational processes rather than direct therapeutic reversal of pathology.

### 4.2 Primitive reflex expression: clinical changes and conceptual considerations

The study documented reductions in the clinical expression of selected PRs during the intervention period, particularly in the CST group. Rather than indicating reflex “abolition” or “deactivation,” these findings more appropriately suggest changes in reflex-related motor responses observed under standardized clinical conditions.

Previous research has reported higher prevalence of retained PRs in children with learning difficulties or attention-related problems compared with typically developing peers (26, 27). For example, persistent ATNR has been associated with attentional and behavioral difficulties in school-aged children (22). However, such associations do not imply the presence of diagnosed neurodevelopmental disorders.

Contemporary neuroscience views PR expression as context-dependent sensorimotor behavior rather than as discrete neurological entities that are either present or absent (3, 25, 28). Variability in reflex expression can occur in typically developing children and does not necessarily indicate neurological immaturity or dysfunction. From this perspective, reduced reflex expression may reflect improvements in postural organization, sensorimotor coordination or task-specific motor control, rather than direct inhibition of reflex circuits. The present findings therefore align with interpretations that clinical expression of PRs may represent subclinical variability within typical development rather than markers of pathology.

### 4.3 Craniosacral findings: interpretation within a debated framework

Changes in palpatory craniosacral findings were observed primarily in the CST group. However, craniosacral constructs, including craniosacral rhythm, mobility and “blocks,” remain controversial and are not broadly accepted within mainstream neuroscience or pediatric neurology (15, 29). Accordingly, these findings should be interpreted cautiously and framed as changes in clinician-perceived tissue or movement characteristics, rather than objective evidence of altered cerebrospinal fluid dynamics or neural function.

While some authors have proposed that gentle manual interventions may influence autonomic regulation, arousal or sensory processing (15, 29), such mechanisms remain speculative and lack robust empirical validation. Previous clinical studies have reported symptomatic improvements following cranial manual therapies in certain conditions, such as migraine (30) and infantile colic (31). Nevertheless, extrapolation of these findings to mechanisms of neurodevelopment should be undertaken with caution.

In this study, craniosacral mechanisms are therefore best conceptualized as potential modulatory influences within a broader dynamic developmental system, rather than as primary causal drivers of neurodevelopmental change. Accordingly, craniosacral findings in this study are best interpreted as changes in clinician-perceived tissue or movement characteristics rather than evidence of specific biological mechanisms. The complete absence of previously identified craniosacral blocks at post-intervention should be interpreted within the context of palpatory subjectivity and limited construct validation. While standardized assessment criteria were applied and assessors were blinded to group allocation, the absence of formally quantified reliability indices may have influenced effect magnitude.

### 4.4 Rhythmic movement training: absence of superiority over placebo

No statistically significant differences were observed between RMT and its placebo condition. Several explanations may account for this finding. First, the placebo intervention may have included active elements (structured movement, therapist interaction, focused attention) that themselves influence motor behavior and test performance. Non-specific therapeutic effects such as expectancy, touch and therapist engagement are well-documented contributors to change in pediatric interventions (32–34). Second, the intensity, frequency or duration of the RMT may have been insufficient to elicit detectable effects beyond placebo. Previous studies reporting benefits of reflex-based exercise programs often employed longer intervention periods or higher session frequency. Third, RMT may preferentially influence specific sensory–motor domains or reflex profiles not optimally captured by the BDI-2 composite score or the reflexes assessed. These findings highlight the importance of carefully designed control conditions and sensitive outcome measures.

### 4.5 Age and baseline characteristics as potential confounders

A statistically significant baseline difference in age was observed between groups. Although small, age is a critical determinant of developmental performance. Developmental changes over relatively short timeframes may partially reflect maturational progression rather than intervention-specific effects. Additionally, qualitative differences in baseline reflex profiles or craniosacral findings may have influenced responsiveness. These factors should be considered potential confounders or moderators.

### 4.6 Integration within contemporary developmental theory

Rather than relying on purely maturational explanations, the present findings are more coherently interpreted within system-based and experience-dependent models of development, including Dynamic Systems Theory and Neuronal Group Selection Theory (2, 20). From these perspectives, manual and movement-based interventions may influence developmental outcomes indirectly by modifying attention, arousal, sensory processing or movement variability, rather than by correcting underlying neurological deficits. Such interpretations avoid pathologizing normal developmental variability and align with contemporary developmental neuroscience.

### 4.7 Limitations and future directions

Several limitations must be acknowledged. First, although mixed-effects models with baseline adjustment were implemented, the presence of baseline imbalance in STNR distribution introduces potential residual confounding. Second, reflex and craniosacral assessments relied on clinical observation and palpation without formal quantification of inter-rater or intra-rater reliability coefficients, which may have influenced effect estimates. Third, the short intervention period precludes conclusions regarding durability of effects. Fourth, potential expectancy and contextual placebo effects inherent to manual and movement-based interventions cannot be fully eliminated despite matched placebo conditions. Fifth, all participants were recruited from a single private educational center, which may limit external validity. Potential clustering effects at the classroom level were not formally modeled, as intervention sessions were delivered individually rather than classroom based. However, shared environmental and pedagogical factors cannot be entirely excluded. Future multicenter studies incorporating hierarchical modeling would strengthen generalizability and allow evaluation of potential school-level influences. Finally, the study population consisted of typically developing children recruited from a single educational center, limiting generalizability and public health extrapolation.

### 4.8 Potential investigator allegiance and bias

The authors have prior clinical and research experience in CST, which may introduce allegiance bias. Placebo controls, standardized protocols, blind assessors and predefined criteria were used to mitigate this risk. Nevertheless, expectancy and contextual influences cannot be fully eliminated and should be considered when interpreting findings. Accordingly, the study should be understood as exploratory, with findings that are hypothesis-generating rather than confirmatory or prescriptive.

## 5 Conclusion

In this randomized controlled trial with baseline-adjusted longitudinal modeling, CST was associated with short-term improvements in standardized developmental assessment scores and reductions in clinically observed primitive reflex expression compared with placebo in typically developing children. Effect sizes were small to moderate, and findings reflect short-term functional modulation rather than demonstrated neurodevelopmental acceleration or structural neurological change. No superiority of RMT over placebo was detected. Given baseline imbalances, subjective outcome components and short follow-up, results should be considered preliminary and hypothesis-generating. Replication using objective measures, quantified reliability metrics, longer follow-up and independent research teams are warranted before clinical or preventive recommendations can be made.

## Data Availability

The raw data supporting the conclusions of this article will be made available by the authors, without undue reservation.

## References

[1] Calvin WH. Neural darwinism. Science. (1988) 240:1802. doi: 10.1126/science.240.4860.1802 17842436

[2] Thelen E Smith LB. A Dynamic system Approaches the Development of Cognition and action. Cambridge, MA: MIT Press (1994).

[3] Hadders-Algra M. Early human motor development: from variation to the ability to vary and adapt. Neurosci Biobehav Rev. (2018) 90:411–27. doi: 10.1016/j.neubiorev.2018.05.009, 29752957

[4] Adolph KE Hoch JE. Motor development: embodied, embedded, enculturated and enabling. Annu Rev Psychol. (2019) 70:141–64. doi: 10.1146/annurev-psych-010418-102836, 30256718 PMC6320716

[5] Zafeiriou DI. Primitive reflexes and postural reactions in the neurodevelopmental examination. Pediatr Neurol. (2004) 31:1–8. doi: 10.1016/j.pediatrneurol.2004.01.012, 15246484

[6] León-Bravo G Cantarero-Carmona I Caballero-Villarraso J. Prevalence of active primitive reflexes and craniosacral blocks in apparently healthy children and relationships with neurodevelopment disturbances. Children. (2023) 10:1014. doi: 10.3390/children10061014, 37371246 PMC10296916

[7] Hickey J Feldhacker DR. Primitive reflex retention and attention among preschool children. J Occup Ther Sch Early Interv. (2022) 15:1–13. doi: 10.1080/19411243.2021.1910606

[8] Pecuch A Gieysztor EZ Telenga M Paprocka-Borowicz M. Persistence of primitive reflexes and associated motor problems in healthy preschool children. Front Neurol. (2020) 11:849. doi: 10.3389/fneur.2020.00849, 29379547 PMC5778413

[9] Johnson MH. Developmental cognitive neuroscience: An introduction. 5th ed. Hoboken, NJ: Wiley (2019).

[10] Pathak A Gyanpuri V Dev P Dhiman NR. The Bobath concept (NDT) as rehabilitation in stroke patients: a systematic review. J Family Med Prim Care. (2021) 10:3983–90. doi: 10.4103/jfmpc.jfmpc_528_21, 35136756 PMC8797128

[11] Zanon MA Porfírio GJM Riera R Martimbianco ALC. Neurodevelopmental treatment approaches children with cerebral palsy. Cochrane Database Syst Rev. (2015) 2015:CD011937. doi: 10.1002/14651858.CD011937

[12] Dempsey A Blomberg K. Rhythmic movement training and primitive reflex integration: a clinical perspective. J Bodyw Mov Ther. (2011) 15:343–52. doi: 10.1016/j.jbmt.2010.08.00221665111

[13] Blomberg K. Rhythmic Movement Training: Integrating Primitive Reflexes for Improved motor Development. Stockholm, Sweden: Blomberg Rhythmic Movement Institute (2015).

[14] Pilat A. "Myofascial therapies". In: Myofascial Induction, vol. 53. Buenos Aires: Editorial Interamericana (2003). p. 1689–99.

[15] Cook AC Egli AE Cohen NE Bernardi K Chae MY Kapalko BA . The neurophysiological effects of craniosacral treatment on heart rate variability: a systematic review of literature and meta-analysis. Cureus. (2024) 16:e64807. doi: 10.7759/cureus.64807, 39156412 PMC11329942

[16] Amendolara A Sheppert A Powers R Payne A Stacey S Sant D. Effectiveness of osteopathic craniosacral techniques: a meta-analysis. Front Med. (2024) 11:1452465. doi: 10.3389/fmed.2024.1452465, 39430589 PMC11487524

[17] Raith W Marschik PB Sommer C Maurer-Fellbaum U Amhofer C Avian A . General movements in preterm infants undergoing craniosacral therapy: a randomised controlled pilot-trial. BMC Complement Altern Med. (2016) 16:12. doi: 10.1186/s12906-016-0984-5, 26758035 PMC4710971

[18] Goddard Blythe S. The well-Balanced child: Movement and Early Learning. London: Orion (2005).

[19] Newborg J Stock JR Wnek L Guidubaldi J Svinicki J. Battelle Developmental Inventory–second edition (BDI-2). San Antonio, TX: Pearson Assessments (2005).

[20] González DA Reséndiz A Reyes-Lagunes I. Adaptation of the BDI-II in Mexico. Salud Mental. (2015) 38:237–44. doi: 10.17711/SM.0185-3325.2015.033, 28936017 PMC5603280

[21] Upledger JE Vredevoogd JD. Craniosacral Therapy. Seattle, WA: Eastland Press (1995).

[22] Gieysztor E Pecuch A Kowal M Borowicz W Paprocka-Borowicz M. Pelvic symmetry is influenced by asymmetrical tonic neck reflex during young children’s gait. Int J Environ Res Public Health. (2020) 17:4759. doi: 10.3390/ijerph17134759, 32630679 PMC7370024

[23] Alamiri B Nelson C Fitzmaurice GM Murphy JM Gilman SE. Neurological soft signs and cognitive performance in early childhood. Dev Psychol. (2018) 54:2043–52. doi: 10.1037/dev0000566, 30265034 PMC6202138

[24] Melillo R Leisman G Mualem R ornai A Carmeli E. Persistent childhood primitive reflex reduction effects on cognitive, sensorimotor and academic performance in ADHD. Front Public Health. (2020) 8:431835. doi: 10.3389/fpubh.2020.431835, 33282806 PMC7706103

[25] Pecuch A Gieysztor E Wolańska E Telenga M Paprocka-Borowicz M. Primitive reflex activity in relation to motor skills in healthy preschool children. Brain Sci. (2021) 11:967. doi: 10.3390/brainsci11080967, 34439585 PMC8394673

[26] McPhillips M Hepper PG Mulhern G. Effects of replicating primary-reflex movements on specific reading difficulties in children: a randomised, double-blind, controlled trial. Lancet. (2000) 355:537–41. doi: 10.1016/S0140-6736(99)02179-0, 10683004

[27] Konicarova J Bob P. Asymmetric tonic neck reflex and symptoms of attention deficit and hyperactivity disorder in children. Int J Neurosci. (2013) 123:766–9. doi: 10.3109/00207454.2013.801471, 23659315

[28] Hadders-Algra M. The neuronal group selection theory: a framework to explain variation in normal motor development. Dev Med Child Neurol. (2000) 42:566–72. doi: 10.1017/s0012162200001067, 10981936

[29] Ceballos-Laita L Ernst E Carrasco-Uribarren A Cabanillas-Barea S Esteban-Pérez J Jiménez-Del-Barrio S. Is craniosacral therapy effective? A systematic review and meta-analysis. Healthcare. (2024) 12:679. doi: 10.3390/healthcare12060679, 38540643 PMC10970181

[30] Arnadottir TS Sigurdardottir AK. Is craniosacral therapy effective for migraine? Tested with HIT-6 questionnaire. Complement Ther Clin Pract. (2012) 19:11–4. doi: 10.1016/j.ctcp.2012.09.003, 23337558

[31] Castejón-Castejón M . Effectiveness of craniosacral therapy in the treatment of infantile colic. Complement Ther Med. (2019) 47:102164. doi: 10.1016/j.ctim.2019.07.023 31780018

[32] Benedetti F. Placebo Effects: Understanding the Mechanisms in Health and Disease. Oxford: Oxford University Press (2008).

[33] Ozpolat C Okcay Y Ulusoy KG Yildiz O. A narrative review of the placebo effect: historical roots, current applications and emerging insights. Eur J Clin Pharmacol. (2025) 81:625–45. doi: 10.1007/s00228-025-03818-6, 40080139

[34] Niazi SK. Placebo effects: neurological mechanisms inducing physiological organic and belief responses-a prospective analysis. Healthcare. (2024) 12:2314. doi: 10.3390/healthcare12222314, 39595511 PMC11593399

