# Supplementary material for: Short-term effects of craniosacral therapy and rhythmic movement training on developmental assessment scores and primitive reflex expression in typically developing children: a randomized controlled trial
Source: Front Public Health. 2026 Mar 26;14:1771040. doi: 10.3389/fpubh.2026.1771040 (PMC13062334; doi:10.3389/fpubh.2026.1771040)
Supplement: Supplementary file 1 [file supplementary_file_1.docx]

# Supplementary Material

Annex 1

**Craniosacral techniques to unblock Craniosacral Blocks (CB)**

**Duramater Balance**: The patient lies on their side with their back to the therapist. The therapist places their hand closest to the skull at the suboccipital level and their other hand more caudally on the sacrum. A downward movement is performed on both bony prominences, with the occiput moving upwards towards the apex while the sacrum moves caudally downwards. When this movement reaches its maximum amplitude, both bones retract and approximate each other through the flexibility and movement of the dura mater. This technique is performed for 3 minutes while the patient is lying on their side.


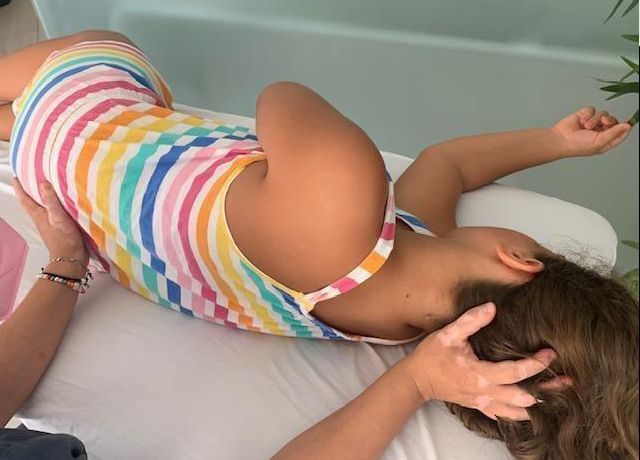


**Sphenoid Bone**: This procedure is performed with the patient lying supine while the physiotherapist, from the cranial region, assesses the compression or decompression at the sphenobasilar junction and the release of horizontal and anteroposterior cerebellar retractions over the greater wings of the sphenoid bone. If there is no synchronization between the left and right sides, it indicates an alteration in sphenobasilar decompression, causing an impact on the pituitary gland and the sella turcica. First phase (compression): The therapist applies light caudal pressure, towards the treatment table, using their thumbs. The pressure must be subtle enough to prevent the fingers from moving across the skin. Second phase (decompression): The physiotherapist's hand positioning is the same as in the previous phase. Now, applying pressure with both thumbs, the physiotherapist performs traction in the opposite direction, that is, upwards, towards the ceiling. Once resistance is reached, this position must be maintained for at least five minutes to achieve complete relaxation. Finally, as in the previous phase, the pressure must be released very gently.


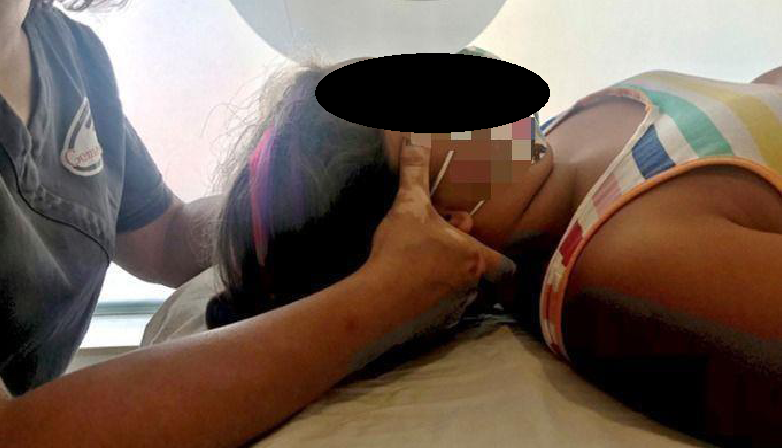


**Parietal Bone**: With the patient in a supine position, the physiotherapist rests their forearms on the treatment table, placing their fingers spread apart on the parietal bones along the squamous (temporoparietal) suture and immediately above the temporal bones. The thumbs are interlaced without touching the skull. An impaction of one or both parietal bones is said to exist when their cranial traction is less pronounced or practically nonexistent compared to the opposite side, due to a caudal impaction. The action is performed in two phases: First phase: compression of the parietal bones (3 to 5 minutes). Second phase: traction or decompression of the parietal bones (1 to 3 minutes).


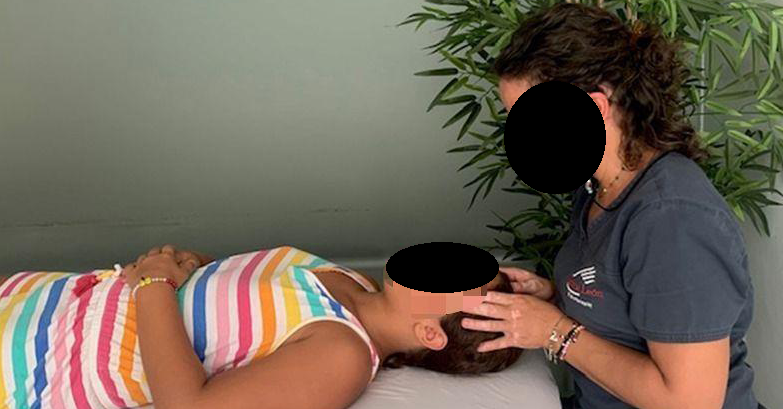


Annex 2

**Rhythmic Movement Training (RMT) to inhibit Primitive reflex expression (PRE)**

**Moro Reflex (RMT):** In Rhythmic Movement Training (RMT), the integration of the Moro reflex is addressed through passive, rhythmic movements that involve gentle flexion–extension patterns of the trunk and symmetrical movements of the upper and lower limbs performed in a slow and repetitive manner. These movements provide regulated proprioceptive and vestibular stimulation, which contributes to reducing hyperexcitability of the brainstem-mediated startle response. Repetition of these primitive movement patterns is thought to promote maturation of vestibular–reticular pathways and enhance autonomic regulation, thereby facilitating a progressive decrease in the activation threshold of the Moro reflex and supporting its cortical inhibition.

**Asymmetric Tonic Neck Reflex (ATNR):** For the asymmetric tonic neck reflex, RMT employs rhythmic movements incorporating alternating head rotation together with slow, symmetrical and crossed-limb motions. These movement patterns provide bilateral sensory input and encourage symmetrical activation of both sides of the body, which is believed to facilitate interhemispheric communication and reduce lateralized reflex dominance. The repetitive nature of these exercises supports gradual cortical inhibition of the ATNR and promotes the emergence of integrated bilateral motor patterns associated with improved postural stability and coordination.

**Symmetric Tonic Neck Reflex (STNR)**: The integration of the symmetric tonic neck reflex in RMT is facilitated by rhythmic movements that simulate early quadruped developmental patterns, combined with gentle cervical flexion–extension synchronized with trunk and limb movements. This approach aims to reinforce coordination between head position and limb activity while promoting dissociation between cervical motion and extremity responses. Through repeated exposure to these coordinated rhythmic sequences, RMT is proposed to strengthen functional connections between the brainstem, cerebellum and motor cortex, supporting the transition from reflex-driven postural control toward voluntary and more mature motor organization.
